# Psychological burden and resilience factors in patients with Alveolar Echinococcosis – A cross-sectional study

**DOI:** 10.1371/journal.pntd.0007082

**Published:** 2019-01-07

**Authors:** Christoph Nikendei, Anja Greinacher, Anastasiya Berkunova, Thomas Junghanss, Marija Stojkovic

**Affiliations:** 1 Department of General Internal Medicine and Psychosomatics, University Hospital Heidelberg, Germany; 2 Section Clinical Tropical Medicine, Department of Infectious Diseases, University Hospital Heidelberg, Germany; Istituto Superiore di Sanità, ITALY

## Abstract

**Background:**

Alveolar echinococcosis (AE) is a parasitic zoonosis resembling malignancy due to its clinically silent infiltrative growth, predominately in the liver. The comorbid psychological burden and fear of disease progression in AE patients have hardly been examined to date. The aim of this study was to evaluate depression, anxiety, quality of life, and fear of disease progression in AE patients.

**Methodology/Principal findings:**

In a cross-sectional study, n = 57 AE patients were invited to report on depression (PHQ-9), anxiety (GAD-7), somatic symptom load (SSS 8), trauma symptoms (PTSS-10), quality of life (SF-12) and on fear of disease progression (FoP-Q-SF) using validated psychometric instruments. Furthermore, attachment style was assessed (RQ-2). N = 47 patients completed the questionnaires (response rate 82.5%). Depression, anxiety, and somatic symptom load were above norm sample means, while physical quality of life was below norm sample means. Existing traumatic symptoms were comparable to those in cancer patients, while fear of disease progression even exceeded cancer patient scores. Patients with a secure attachment style showed less pronounced psychological burden than patients with other attachment styles. Adequate, guideline-based depression and anxiety treatment was very rarely installed.

**Conclusion/Significance:**

The present study revealed remarkable levels of psychological burden in AE patients. In our study sample, we discovered high depression and anxiety levels, a significant reduction of physical quality of life, and fear of disease progression. These results show how important it is for AE patients to be thoroughly assessed with regard to psychological symptoms and mental disorders so that those in need can receive sufficient psychosocial support and treatment according to official guidelines.

## Author summary

Alveolar echinococcosis (AE) is a parasitic zoonosis resembling malignancy due to its clinically silent infiltrative growth, predominately in the liver. For some somatic diseases, comorbid psychological burden predicts the course of disease. However, as far as we are aware, little is known about comorbid psychological burden and fear of disease progression in AE patients. In a cross-sectional study, AE patients were invited to report on depression, anxiety, somatic symptom load, symptoms of posttraumatic stress, quality of life, and their fear of disease progression. Furthermore, we assessed the patients’ attachment style. The present study revealed that depression, anxiety, and somatic symptom load were above norm sample means. According to our findings, symptoms of posttraumatic stress were comparable to those in cancer patients and fear of disease progression even exceeded scores found in cancer patients. Overall, psychological burden was less pronounced in securely attached patients. AE patients rarely received treatment for their depression or anxiety disorders according to German guidelines. Thus, our results show how important it is that AE patients are thoroughly assessed with regard to psychological symptoms and mental disorders, in order to give psychosocial support and supply treatment to those in need.

## Introduction

Alveolar echinococcosis (AE) is a parasitic zoonosis. The life cycle of *Echinococcus multilocularis* predominately runs between foxes and rodents [1]. Humans are generally infected accidentally. The parasite’s target organ is the liver with an infiltrative clinically silent growth pattern, and—in advanced disease—local and distant metastases. For malignancy, AE, thus, is a main differential diagnosis [2]. In early stages of the disease, the lesion(s) is (are) resected; in advanced stages, further growth is suppressed with benzimidazoles and patients are closely monitored for side effects and complications. Currently, benzimidazoles are the only available drugs to treat AE. It is crucial to note that benzimidazoles merely have a parasitostatic effect and cannot eradicate the parasite in most cases. Due to its nature, AE may be cause of psychological impairment. So far, only one study [3] has focused on the psychological burden of AE patients and discovered that AE patients have a reduced mental quality of life. As far as we are aware, there has not been any other research on comorbid psychological burden and fear of disease progression (FoP) in AE patients.

Compared to persons who either have a physical or mental health problem, patients who present both a somatic illness *and* a psychological comorbidity show dramatically reduced outcomes in all important dimensions of health: they have higher mortality rates [4–7] as well as higher levels of symptom burden and impairment in any medical disorder index [8]. In addition, health care costs are significantly higher in patients with both chronic conditions and comorbid mental disorders [9], although the increased costs are mainly due to medical, and not psychosocial care [10].

Depression and anxiety disorders are the most frequent comorbidities of somatic diseases. Depressive disorders are predominantly characterised by persistent depressive mood, loss of interest or pleasure, and loss of energy [11]. With a life-time prevalence of up to 24% for major depressive episodes [12, 13], which puts them among the most common psychiatric disorders. More common in females, depressive disorders are associated with a significant impairment of quality of life [14] and with high psychological strain, including suicidal behavior [15, 16].

Anxiety disorders, comprising general anxiety, panic or phobic disorders, are also highly prevalent in the general population [17]. They lead to avoidance behavior, social withdrawal, and are highly resistant to spontaneous recovery without specialized treatment [18]. Although not defined as an anxiety disorder, FoP (or fear of recurrence) is a well-known phenomenon which has mainly been researched in cancer patients. FoP is defined as an appropriate, rational response to a real physical threat and somatic treatment, which can become dysfunctional, affecting well-being, quality of life, and social functioning [19].

So far, resilience factors protecting affected persons from developing comorbid psychological disorders have not been investigated in AE patients. However, attachment style has been widely shown to be an important resilience factor [20]. Attachment style is defined as a psychological motivational system which stores perceptions, interpretations, and expectations of interpersonal interactions. This system is shaped by repeated biographical interaction experiences and results in an enduring “internal working model” which is activated in situations of need, illness, or other experiences of excessive demand [21–23]. We can distinguish four different attachment styles, namely the secure, the anxious-ambivalent, the anxious-avoidant, and the disorganized attachment style. All four types are composed of the model of the self (positive vs. negative) and the model of others (negative vs. positive). The secure attachment style is categorized as the most mature way in which individuals respond to their own interactional needs and their partners’ requirements [24].

The aims of the current cross-sectional study were: (1) to assess depression, anxiety, somatic symptom burden, symptoms of posttraumatic stress, quality of life, and FoP in AE patients; (2) to examine differences in psychological burden based on gender, previous surgical interventions, medical leave and the duration of the illness; (3) to explore the relationship between FoP and attachment style. We hypothesised that (i) depression, anxiety, somatic burden, and trauma symptoms would be more pronounced compared with the general population. We thought that the quality of life would be lower in AE patients than in the general population, while FoP would be greater compared to FoP in specific groups of cancer patients (patients with malignant melanoma, prostate cancer, or breast cancer). Further, we estimated that (ii) patients of female gender, patients without curative surgery, and patients with a longer duration of illness would present more symptoms of psychological burden. We hypothesized that medical leave due to AE would be negatively correlated with psychological burden. Lastly, we thought that (iii) psychological burden (depression, anxiety, somatic burden, trauma symptoms, and FoP) would be elevated in patients with insecure attachment styles.

## Methods

### Study design, study population, and ethical considerations

The study is cross-sectional. A cohort of AE patients that were diagnosed following the diagnostic criteria [25] of the expert consensus and consecutively recruited during regular patient contacts at the clinic for echinococcosis at the Section of Clinical Tropical Medicine, University Hospital Heidelberg. Since 1999, 158 patients with AE have been diagnosed, treated, and followed up at the Section of Clinical Tropical Medicine. The clinic for echinococcosis is run in cooperation with the Departments of Diagnostic and Interventional Radiology, Gastroenterology, Parasitology, and Surgery and is a national clinical reference center for echinococcosis. In total, 57 AE patients who were attending the clinic for echinococcosis between June 2016 and May 2017 were informed and invited by the physician in charge to participate in the study. There were no exclusion criteria. All participants provided an informed consent and could withdraw their participation without any disadvantage. All the analysed data were anonymized. However, if requested before enrolment in the study, participants received a personalized summary and detailed explanation of their test results. Furthermore, all participants were invited to receive psychological consultation at the Department of General Internal Medicine and Psychosomatics. The study was approved by the ethics committee of the University Hospital Heidelberg (ethics application no. S-232/2016). The study was conducted in accordance with the Declaration of Helsinki (most recent version: Fortaleza, Brazil, 2013).

### Survey instruments

#### Sociodemographic data

The following sociodemographic data were collected: age, gender, nationality, marital status, number of children, place/country of birth, educational qualification, profession, and further qualifications. Additionally, the number of periods of medical leave due to echinococcosis, engagement in psychotherapy, and the use of psychiatric medication was documented.

#### Patient Health Questionnaire (PHQ-9)

Depression was assessed using the depression module of the Patient Health Questionnaire (PHQ; [26], German version [27]). The severity of depressive symptoms is determined by the sum score, which can range between 0 and 27. There are four categories of depression: minimal (sum score 1–4), mild (5–9), moderate (10–14), and severe (15 or above) [28]. In a representative German norm sample, the mean sum score for depression was M = 3.6 (SD = 4.1) [29]. The internal consistency in our data is Cronbach's α = .89.

#### Generalized Anxiety Disorder Scale (GAD-7)

The anxiety module of the Patient Health Questionnaire, the Generalized Anxiety Disorder Scale (GAD-7), was used to assess symptoms of generalized anxiety ([30]; German version [31]). The sum score ranges from 0 to 21. The final result gives four categories of the severity of anxiety symptoms: minimal (sum score 1–4), mild (5–9), moderate (10–14), and severe (15 or above) [30]. In a study with a representative population sample, the mean sum score of the GAD-7 was M = 2.9 (SD = 3.4) [31]. The internal consistency in our data is Cronbach's α = .86.

#### Posttraumatic Stress Scale (PTSS-10)

The PTSS-10 [32] is a short screening instrument for Posttraumatic Stress Disorder (PTSD) and is based on the diagnostic criteria according to the DSM. The scale includes 10 items which comprise typical symptoms due to trauma (re-experience, avoidance, and arousal). On a scale of 4 (0 = not at all to 3 = often) the respondents estimate how much they suffered from the given symptoms during the last seven days. The total PTSS-10 score is calculated by adding the item scores, which gives an overview of the current symptom load. Maercker [32] specifies a cutoff value of 12 for the suspected diagnosis of PTSD. The internal consistency in our data is Cronbach's α = .89.

#### Somatic Symptom Scale (SSS-8)

The SSS-8 [33] was developed as a short form of the frequently used PHQ-15 [28]. It consists of eight items and can be used to assess the severity of current physical strain. Excellent item characteristics and good reliability values were shown in a representative German population sample with N = 2510 subjects [33]. Hence, the questionnaire short form is a valid and reliable instrument. Sum scores range between 0 and 32 and the overall score concerning the somatic symptom load can be interpreted as follows: 0–3 minimal, 4–7 low, 8–11 medium, 12–15 high, 16–32 very high. The internal consistency in our data is Cronbach's α = .84.

#### Short Form Survey (SF-12)

The German version [34] of the SF-12 [35] is the short form of the 36-item Short Form Survey (SF-36) and measures the physical and mental quality of life. In general, quality of life is understood as the patient’s perception of his or her functionality and well-being in everyday life. The SF-12 consists of two of the eight scales of the SF-36, namely the Physical Component Summary (PCS) and the Mental Component Summary (MCS). The sum score ranges from 0 to 100. 0 stands for a very low quality of life and 100 represents the highest (optimal) quality of life.

#### Fear of Disease Progression Questionnaire–Short Form (FoP-Q-SF)

The FoP-Q-SF is the short form (German version PAF-F-KF; [36]) of the Fear of Disease Progression Questionnaire [37] and consists of 12 items. With this questionnaire, investigators evaluate whether and how strongly patients who have been diagnosed with a serious illness are afraid of the further development of their disease. The self-report tool comprises five subscales (affective reactions, partnership/family, job, loss of autonomy and coping with anxiety). The FoP-Q-SF was validated with a sample of 1083 patients with breast cancer and is recommended as a reliable assessment instrument for research and clinical care [36]. In a study by Engst-Hastreiter et al. [38], the cut-off value for heavily burdened patients is set at >36. The internal consistency in our data is Cronbach's α = .85.

#### Relationship Questionnaire (RQ)

The Relationship Questionnaire (RQ; [39, 40]) was used to assess the attachment style regarding the self and others. Attachment style is defined as combining both the model of the self (positive / negative) and the model of others (positive / negative). The RQ differentiates between four different attachment styles: secure, anxious-ambivalent, anxious-avoidant, and disorganized. The questionnaire was designed to assess continuous ratings. In our study we used the scores of the model of the self and the model of the others. Standard values are not available.

### Analysis

All data were coded and analysed using SPSS (version 24). Missing values were not included in the calculation. The raw data are displayed by showing mean and standard deviations. Due to a sample size above 30, we assumed a normal distribution in our data [41]. We chose a significance level of 5% (two-sided). We compared the group of study participants with representative norm samples and other patient groups by using the Welch test (tested two-sided) due to the lack of homoscedasticity. For within-group comparisons, we used the Welch test (tested two-sided) in order to describe differences in psychological burden (depression and anxiety scores, somatic symptoms, posttraumatic stress, quality of life, and FoP) in regard to an individual’s gender, previous surgical intervention, and medical leave due to echinococcosis. We used Pearson’s correlation to assess differences in psychological burden in connection with the duration of the disease, and the number of years since the initial diagnosis. In order to investigate the correlation between the ‘model of self’ and the ‘model of others’ and levels of psychological burden, we also applied Pearson’s correlation coefficients.

## Results

### Response rate and sample description

47 out of 57 AE patients completed the questionnaires, equalling an overall response rate of 82.5%. Table 1 depicts detailed sample characteristics.

**Table 1:** Assessed alveolar echinococcosis patients’ sociodemographic characteristics (n = 47).

	n	%
Gender		
Male	18	38.3
Female	29	61.7
Age		
Mean (SD)	58.21	(17.58)
Citizenship		
German	45	95.7
Russian	1	2.2
Croatian	1	2.2
Marital status		
Married	27	57.4
In a relationship	6	12.8
Widowed	9	19.1
Single	1	2.1
Time since initial diagnosis		
Mean (SD)	5.55	(3.95)
Attachment		
Model of self: Mean (SD)	4.61	(4.29)
Model of others: Mean (SD)	1.16	(4.37)
AE status		
Critical	22	46.8
Uncritical	15	31.9
Cured	10	21.3
Treatment		
Currently in psychotherapy	2	4.3
Psychotherapy in past	12	25.5
Psychiatric medication	2	4.3
Medical leave / retirement		
No medical leave	28	59.6
Medical leave	16	34.0
Retirement	1	2.1

* Russian: n = 1, Croatian: n = 1;° missing: indicated other n = 4 (9%); # missing: n = 3 (6%)

AE clinical status: ‘critical’ = AE lesion(s) non resectable, AE progression not well controlled by benzimidazoles, significant adverse benzimidazole effects and/or complications, such as cholangitis, biliary system obstruction, compression of blood vessels or adjacent organs, distant metastases; ‘uncritical’ = AE lesion(s) non resectable, AE progression well controlled by benzimidazoles; ‘cured’ = patient free of disease at long term follow-up after resection of AE lesion(s) and two years of postoperative benzimidazoles.

### Psychological comorbidity

Table 2 displays the psychological comorbidity of the assessed AE patients. The study participants showed a significantly higher level of depressive symptoms (p = .015; 31) and general anxiety symptoms than participants of a representative general-population survey (p = .015; [29]). Table 3 presents the distribution of patients classified by rating their depression and anxiety symptoms as minimal / mild / moderate / severe. In the PTSS-10 questionnaire, 46 AE patients (97.9%) scored higher than 12 points, implicating the need for further diagnostic clarification regarding posttraumatic stress disorder. The mean score of the PTSS-10 in AE patients shows that their posttraumatic stress is higher than posttraumatic stress in patients currently undergoing aftercare of malignant melanoma (p = .045; [42]). Additionally, somatic ailment was significantly more pronounced in AE patients than in the general population (p < .001; [33]). Participants reported a significantly lower physical quality of life compared to a representative general population survey (p < .001), but showed an average mental quality of life (p = .110, [43]). Furthermore, AE patients (female and male) showed a significantly higher FoP compared to patients with prostate cancer (p < .001; [44]). A sum score on the FoP-Q-SF above 36 indicates a high level of fear [38]. In the current study, 18 AE patients (38.3%) reported levels of 36 or above this cut-off score.

**Table 2:** Symptom scores of psychological assessment in alveolar echinococcosis patients.

Echinococcisis sample				Norm sample / patient sample						
	N	M	SD		N	M	SD	t		df
PHQ-9	47	5.33	5.29	NS:	2066	3.6	4.08	2.229	*	47
GAD-7	45	4.35	4.17	NS:	5036	2.95	3.41	2.247	*	44
PTSS-10	47	26.53	13.44	PS^a^	70	22.27	12.9	1.708		960
SSS-8	45	9.91	6.48	NS:	2510	3.23	3.96	6.893	**	44
MCS	43	47.95	10.71	NS:	21248	50	10	-1.253		42
PCS	43	43.23	11.04	NS:	21248	50	10	-4.015	**	42
FoP-Q-SF	47	34.63	11.03	PS^b^:	44	26	9.2	4.043	**	87
				PS^c^:	76	31	9	1.903		83

NS = norm sample; PS = patient sample; N = sample size; M = mean; SD = standard deviation

* p < .05

** p < .01

^a^ = patients with malignant melanoma

^b^ = prostate cancer patients

^c^ = breast cancer patients

**Table 3:** Distribution of depression (n = 47) and anxiety scores (n = 45) categorized as minimal / mild / moderate / severe.

	scores	n	%
Symptoms of depression			
Minimal	0–4	30	63.8
Mild	5–9	8	17.1
Moderate	10–14	5	10.6
Severe	15–27	4	8.5
Symptoms of anxiety			
Minimal	0–4	28	59.6
Mild	5–9	8	17.0
Moderate	10–14	9	19.1
Severe	15–21	-	-

### Impact of gender, previous surgical interventions, medical leave, and duration of disease on psychological comorbidity

We revealed a difference connected to patients’ gender concerning the following three aspects of psychological comorbidity: Male participants (M = 2.80, SD = 2.34) showed lower scores in the PHQ-9 (depression) than female participants (M = 6.91, SD = 6.00; Welch test: t(39.525) = 10.901, p = .002); male participants (M = 52.54, SD = 9.58) presented a higher psychological quality of life compared to female participants (M = 44.95, SD = 10.50; Welch test: t(36.562) = 5.974, p = .019); regarding FoP, male participants had lower scores (M = 30.89, SD = 7.89) than female participants (M = 36.97, SD = 12.14; Welch test: t(44.862) = 4.323, p = .043).

For further analysis, AE patients were divided into two groups, namely cured patients (definition see above; n = 10) and non-cured patients (critical and uncritical patients; definition see above; n = 37). Cured patients who had had surgical intervention showed no difference in psychological comorbidity (depression, anxiety, quality of life, somatic symptom burden, posttraumatic stress symptoms, and FoP; all p > .05) compared to non-cured patients. Study participants (n = 16) who reported to have had at least one period of medical leave from work due to AE showed significantly higher FoP (M = 38.53, SD = 11.84) than participants who did not have any medical leave (M = 31.14, SD = 9.49; Welch test: t(26.084) = 4.593, p = .042). No significant correlation was found between the number of years since the initial diagnosis had been made and the psychological burden (depression, anxiety, somatic symptom burden, posttraumatic stress symptoms, FoP and quality of life; r = -.17 - .08; all p > .05).

### Attachment as resilience factor

Table 4 presents attachment styles of AE patients and their correlation with psychological burden. Attachment of the self correlates significantly with anxiety (r = -.319, p < .05) and trauma symptoms (r = -.331, p < .05) and FoP (r = -.334, p < .05). Attachment to others correlates significantly with depression (r = -.305, p < .05), anxiety (r = -.372, p < .05) and trauma symptoms (r = -.300, p < .05). This suggests that AE patients who are more securely attached may be less likely to develop symptoms of depression, anxiety disorders, FoP, or trauma.

**Table 4:** Attachment styles of AE patients (n = 44) and the correlation with RQ scores.

Echinococcosis patients, N = 44							
			Correlation with				
	M	SD	PHQ-9	GAD-7	SSS-8	PTSS-10	FoP-Q-SF
RQ self	4.61	4.29	-.149	-.319*	-.069	-.331*	-.344*
RQ other	1.16	4.37	-.305*	-.372*	-.295	-.300*	-.032

* p < .05

## Discussion

Our study assessed the psychological burden of AE patients in a cross-sectional design using established psychometric instruments. Our main findings show that symptoms of depression and anxiety exceeded corresponding symptom levels of representative norm samples. Furthermore, our data suggests that over 90% of AE patients suffer from symptoms of posttraumatic stress disorder. Overall, the somatic symptom load was very high. In addition, AE patients’ physical quality of life was significantly lower compared to a norm sample, while their mental quality of life proved to be average. A small correlation coefficient between a secure attachment style and lower levels of depression, anxiety, trauma symptoms, and FoP indicates a possible connection. Our results support the outcomes of previous studies which assessed the comorbid psychological burden in AE patients [3] and thereby have broadened the general knowledge on AE, especially pertaining to FoP.

With regard to depression, our data suggest that on average, AE patients suffer from mild to moderate depression. This is comparable to other patient groups with somatic diseases who display comorbid depression [45–47]. Overall, 9 out of the 46 examined AE patients reported moderate or severe depression levels, which entail severe emotional distress and suicidal ideation [15, 16]. These disorders can be treated effectively: according to current guidelines, moderate depression should be treated with psychotherapy or antidepressants, while severe depression requires a combination of both [48]. Importantly, only one of the study participants suffering from depression was currently being treated with psychotherapy and an antidepressant. Another participant was treated with psychotherapy and did not show any signs of depression (any more). Previous studies have shown that somatic patients with comorbid depressive symptoms have a reduced adherence to medical therapy [49–52] and that depression can interfere with treatment and recovery from AE. This may contribute to the fact that patients who have both a somatic and a mental disease show higher levels of symptom burden [53], a higher mortality [4–6], and produce increased health care costs [9]; the latter aspect is mainly attributable to medical, and not to psychosocial care [10]. Despite these facts, there are no studies focusing on depression in AE patients and the role of depression in the development and progression of AE. Our findings further highlight the importance to examine these connections.

Our evaluation of general anxiety symptoms in AE patients gave us comparable results to our outcome regarding depression: study participants presented higher levels of anxiety than representative norm samples. Nine out of the 46 assessed AE patients reached or exceeded the cut-off score, which implies that they have clinical relevant anxiety disorders requiring treatment [54]. Posttraumatic stress was assessed with the posttraumatic stress symptom scale (PTSS-10). Our findings indicate that AE patients may have a high prevalence of posttraumatic stress disorder. However, future studies should focus on evaluating these interesting, preliminary results with well-established instruments that allow a more thorough examination of posttraumatic stress disorders according to DSM criteria, e.g. ETI [55] or PDS [56]. In line with official therapy guidelines, the most effective treatment options for many anxiety disorders are psychotherapy or psychopharmacological treatment [48]. However, only two out of nine AE patients with high scores in the GAD-7 were currently undergoing psychotherapy. None of the patients with high anxiety scores was on psychiatric medication. This is of concern since untreated anxiety disorders show a high persistence of symptoms that may prevail for years [20].

Additionally, the assessed AE patients suffered from a high somatic symptom load, reflected in a low physical quality of life. The subjective physical burden was very high and subsequent constraints on every-day functionality were perceived as impeding. AE patients presented a great FoP, which could be attributed to the following factors: the diagnosis itself with the consequence of physical impairment, being aware of the serious prognosis and outcome, and having to live with an ‘eerie’, parasitic foreign body. According to our results, AE patients show higher levels of FoP than patients suffering from malignant melanoma or prostate cancer. Furthermore, the study participants who presented especially high levels of FoP also reported longer periods of medical leave due to AE.

Our results indicate that certain attachment styles may serve as resilience factors to psychological burden (depression, anxiety, trauma symptoms, and FoP). Thus, patients with an insecure attachment style might be more likely to develop symptoms of psychological burden. This is congruent with previous studies [57–59]. In our research project, however, the correlation coefficients are quite small and would have to be confirmed in further research.

The alarming fact that the AE patients of our study seemed to have especially high prevalence rates of depression, anxiety, posttraumatic stress, and physical burden, but were rarely treated for these comorbidities, highlights how urgent further research with the specific focus on psychological burden would be. Hereby, it might be important to keep in mind that female AE patients seem to be more liable to suffer from depression and to report a lower quality of life. Furthermore, a higher FoP in AE patients is associated with longer periods of medical leave which makes the issue of proper treatment especially relevant. In our opinion, it would be straightforward to implement screening for AE patients using established instruments to cover the most common psychological and psychiatric disorders. If a patient is screened positive, subsequent psychosomatic / psychiatric consultations could be arranged.

### Limitations

Even though our research project has the great advantage of being the first study to investigate the psychological burden and quality of life in AE patients, our findings are limited by the small number of participants. Therefore, our study sample differs considerably to norm samples. Nevertheless, this investigation constitutes the largest research on the psychological burden, quality of life, and FoP in AE patients. Our results are further limited by the aspect that all dependent variables were assessed by self-report measures. Furthermore, it is important to note that our outcome may not be generalizable due to the fact that we were only able to discover a connection between the exhibited psychological symptoms and the personality factor attachment style in a cross-sectional design.

### Conclusions

The present study revealed pronounced psychological burden in AE patients in terms of high levels of depression and anxiety symptoms, a significantly reduced physical quality of life, and high FoP. Our results show how important it is that AE patients are thoroughly assessed with regard to psychological symptoms and mental disorders screening so that sufficient psychosocial support and treatment can be offered to patients in need. Further research should address the long-term development of psychological burden in AE patients, i.e. using follow-up studies to investigate the overall influence of psychological burden on the course of the disease and the impact of sufficient therapeutic support on the disease outcome.

## Supporting information

S1 STROBE Statement (DOCX)

S1 AE Data (XLSX)
